# Origami Expert

**DOI:** 10.1038/s44172-023-00063-6

**Published:** 2023-04-10

**Keywords:** Engineering

## Abstract

Dr. Robert Lang describes how he approaches building origami structures and offers thoughts on the future of origami research.

Dr Robert J. Lang is a full-time origami artist and consultant. His research contributions range from fundamental studies into folding techniques, to technologies for micro scale robotics and large-scale deployable structures for space applications. Here we explore with Dr Lang the connections between art, science and mathematics in origami design, as well as future fundamental and practical challenges for the field.

**Figure:**
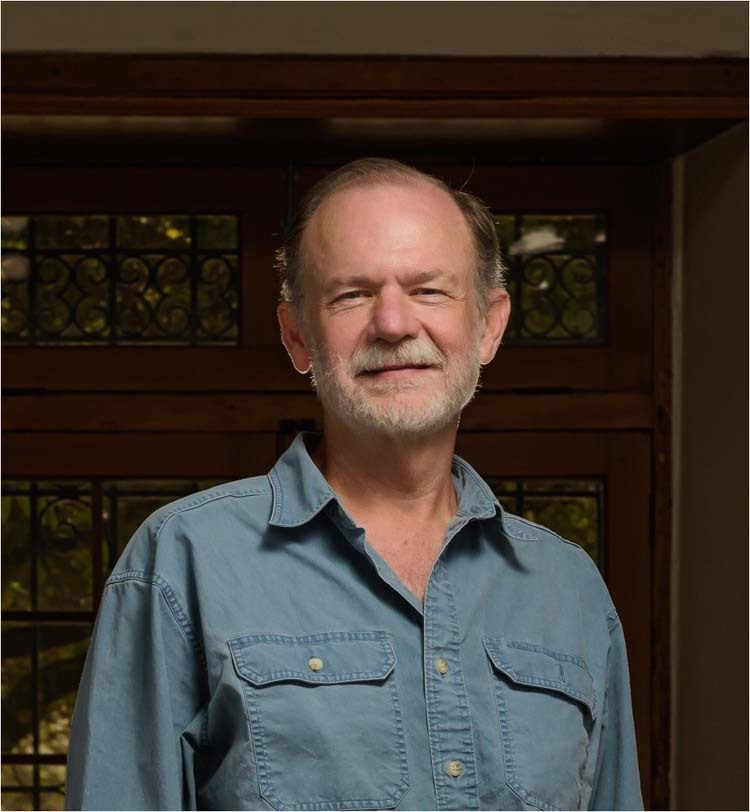
Photograph by Oscar Osorio

1. Tell us about the first time you encountered origami.

I first encountered origami at age six, in the form of instructions for four of the traditional Japanese designs in a craft book. It looked like something fun to try, and it turned out to be so. One of the appealing things was that I didn’t need anything more than a sheet of paper and my hands, and paper was practically free. But also, there was a low barrier to experimentation; once I’d folded the thing according to the instructions, I could try modifications and changes, and if they didn’t work out, just throw away the paper, grab another sheet, and try again.

2. Does your formal academic background in laser physics influence or inform your approach to origami science and design?

It most definitely does. My speciality in lasers was theory: developing mathematical models of the devices and phenomena I was interested in, and then using the tools of mathematics to gain knowledge or provide guidance in their designs. It felt like origami was subject to the same sorts of laws that lend themselves to mathematical description. And so I began to try to use the tools of mathematics and computation that I used in my laser work to describe origami, with the goal of improving my ability to obtain what I wanted in my designs. And it turned out to work amazingly well!

3. Tell us about your favourite piece or pieces from your Portfolio.

My favourite piece is usually my most recent design. Sometimes it’s a very complex design. Sometimes, it’s something fairly simple, but that explores a particular aesthetic quality or geometrical principle. An example of the former is my “Flying Crane, Opus 563” which was recently featured in the February 2023 issue of *National Geographic* Magazine.

**Figure:**
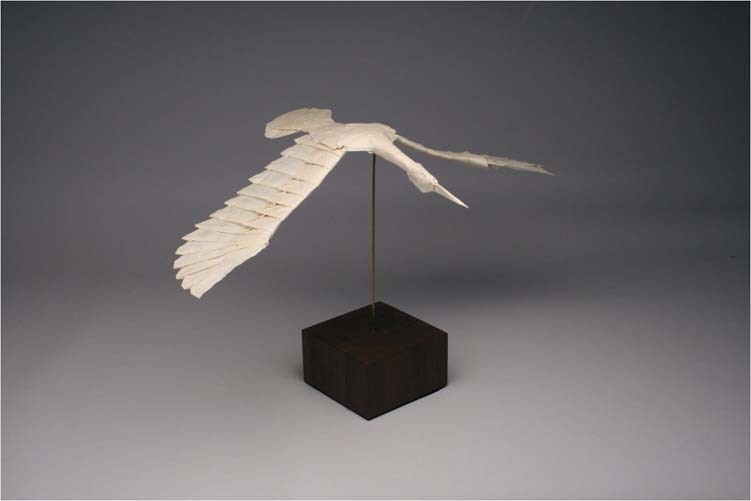
Flying Crane, Opus 563, Robert J. Lang

4. How do you combine artistic intuition with mathematical insight in your designs?

On the timeline of my design, intuition came first. After folding many different figures from instructions in various books and a lot of exploratory folding (“let’s see what happens if I try this”), I began to see patterns in how to obtain different shapes and/or combinations of shapes, so that when I set out to design something, I could draw upon those patterns. I call that intuition because it wasn’t something done according to an algorithm; it was a case of looking at the problem and having a possible solution come to mind. But there’s limits to what intuition can do. If the problem is complicated—like an insect with many legs, wings, and antennae—intuition can come up short.

But some of those intuitive ideas can be described mathematically. For example, if you need a flap that is X cm long, then the tip of that flap must be at least X cm from everything else on the unfolded paper. That notion can be translated into an explicit mathematical inequality that relates the coordinates of the flap tip (in the unfolded paper) to those of other flap tips. When you have a lot of flaps, there’s a lot of relationships to keep track of, which is hard for intuition. But expressed mathematically, it becomes a lot of equations, and we have many ways mathematically and computationally to handle large numbers of equations.

The thing to always keep in mind, though, is that mathematics can only help with structural problems. Art goes beyond pure structure: art is all about aesthetics. So we still have to rely on artistic intuition to address the problem of “what structure do I want that gives the aesthetic goal that I’m after?” We can then use math to help obtain that structure.

5. What do you see as the grand challenges in origami folding yet to be achieved?

There’s a couple. On the mathematical side, the theory of curved folding is seeing a lot of attention these days, but the tools for designing curved folds are still fairly rudimentary. On the artistic side, while historically, representational forms received the most focus (and still do today), the field of purely geometric origami still seems to me to be relatively unexplored, but burgeoning sub-fields like origami tessellations show that there’s a lot of potential there.

6. Many practical applications of origami require deployability or reconfigurability. What are the particular design challenges to creating multistable foldable systems?

Mathematical challenges include accommodating non-ideal folding behaviour. For example, real world folds are only an approximation of the revolute joints by which they are often modelled. And then there are problems associated with material thickness, and materials properties—such as plastic deformation, shearing, creases rolling through the material—that are rarely included in simple design models. At a higher level, for multistable folding forms, we would like to control the number of degrees of freedom (DoF) in multi-configurable structures: things that have many panels and many hinges. The number of attainable DoF in a folding structure with many panels is often “zero,” “one,” or “many.” We’d like to be able to specify “this object with many moving parts has exactly N degrees of freedom in its folding motion”—and of course, have those possible motions be the ones that we need in the application.

7. Are there engineering disciplines which could benefit from origami approaches and which have not been explored much as yet? How can you imagine origami could be applied in these fields?

I’d say that any discipline where mechanical engineering plays a role has the potential to benefit from origami, because origami structures and mechanisms are fundamentally mechanical.

8. Finally, origami techniques have exploded across various technology fields in recent years. Do you have words of caution or insight for engineers who are interested in exploring the practical application of origami and kirigami in their fields?

My primary word of caution is to recognise that origami—or rather, folding (origami interpreted broadly) is a tool, not a magic bullet. Sometimes an old-fashioned linkage from the early days of the Industrial Revolution will do the job just fine!

*This interview was conducted by Rosamund Daw, Chief Editor, Communications Engineering.*

